# An Investigation into the Role of Coping in Preventing Depression Associated with Perfectionism in Preadolescent Children

**DOI:** 10.3389/fpubh.2015.00190

**Published:** 2015-08-07

**Authors:** Silja M. Dry, Robert Thomas Kane, Rosanna M. Rooney

**Affiliations:** ^1^Curtin University, Perth, WA, Australia

**Keywords:** middle childhood, perfectionism, coping, depression

## Abstract

The relationships between self-oriented and socially prescribed perfectionism (SPP) and maladaptive and adaptive coping strategies and their collective impact on depression symptoms were examined in the context of a randomized controlled universal trial of the *Aussie Optimism Positive Thinking Skills Program*. Five hundred and forty-one children aged 8–12 completed a battery of self-reports, of which responses for measures of depression symptoms, perfectionism, and coping strategies were examined for the purposes of this study. Structural equation modeling tested whether coping mediated the effects of perfectionism on depression. Results indicated that SPP had both a direct and an indirect relationship with depression symptoms through a moderate association with maladaptive coping. Implications for prevention of depression were discussed and recommendations for future research were proposed.

## Introduction

Perfectionism or striving for flawlessness is considered an adaptive trait in high achievers and elite athletes as it drives the individual to succeed and as a result contributes positively to mental well-being by improving life satisfaction and self-esteem (1–4). However, as a growing body of research has established, perfectionism may be maladaptive when motivated through a fear of failure, compelling individuals to engage in perfectionistic behavior to avoid failure (5, 6). Individuals with maladaptive perfectionism traits tend to engage in critical self-evaluation against a rigid set of unrealistically high expectations, leading to dichotomous thinking, rumination over mistakes, procrastination, increased stress, dysphoria, and performance dissatisfaction (7, 8).

If follows that maladaptive perfectionism may contribute to the development and maintenance of depression, a clinical disorder that may manifest during adolescence (9–11). As perfectionism traits may emerge at an early age in childhood, research into the interactions with pre-clinical symptoms of depression in children could potentially inform preventive strategies (12).

In examining the relationship between maladaptive perfectionism and depression in children, researchers commonly utilize the Child and Adolescent Perfectionism Scale (CAPS) developed by Flett colleagues, an adaptation of the Multidimensional Perfectionism Scale (MPS) devised to assess intra and interpersonal dimensions of perfectionism in adult populations (13). The measure like its adult counterpart differentiates between self-oriented perfectionism (SOP), the striving for, and critical self-evaluation against self-imposed excessively high standards; and socially prescribed perfectionism (SPP), the striving for, and critical self-evaluation against, the perceived expectation of others (Flett et al., Unpublished)^1^. However, the CAPS omits the “other oriented” perfectionism dimension, which refers to perfectionism being imposed onto others as Flett et al. (Unpublished)^1^ found no developmental evidence to support this factor. Studies with adolescents demonstrated robust associations between symptoms of depression and both SOP and SPP in the context [e.g., Ref. (14–17)], generally replicating findings in adults [see Ref. (9)], while research in younger samples has been limited and with mixed results. Nobel et al. (18) observed that only SOP was significantly correlated with depression in 8- to 11-year-old children identified at-risk for depression or anxiety. This was inconsistent with earlier findings by Huggins et al. (19) who observed that only SPP was associated with a clinical diagnosis of depression in a group of indicated preadolescents (10–11 years). This discrepancy may be due to a potential floor effect in the Nobel et al. study with mean SPP scores almost 2 SDs lower than those reported by Huggins et al. Furthermore, Nobel et al. applied a reduced version of the CAPS, potentially limiting detection of a significant interaction between SPP and depression, as well as its comparability with other studies. Additional research with a larger sample of preadolescents is therefore needed to clarify the nature of association between depression and perfectionism in children.

In examining relationships between perfectionism and depression in adults, researchers have observed that individuals with high levels of perfectionism tend to apply ineffective coping strategies to manage stressful events (20, 21). Coping strategies are considered effective and adaptive when behavioral and cognitive processes include active emotion regulation, constructive thinking, and problem solving techniques to reduce stress (22); and they are considered ineffective or maladaptive when comprising avoidant cognitive and behavioral strategies, which tend to be associated with poorer psychological outcomes (23, 24). Researchers have found that coping is often context driven and depends on the initial appraisal of the situation by the child and their coping repertoire, which becomes more diversified and sophisticated with development (25, 26).

Research has established that adults with high levels of maladaptive perfectionism, specifically SPP, are more likely to apply maladaptive coping (MCOP) and less likely to apply adaptive coping (21, 27). Research on a sample of maltreated adolescents has indicated similar patterns of associations with Flett et al. (28) observing that SOP was correlated with problem solving (an adaptive coping strategy), while SPP was negatively correlated to problem solving and positively related to avoidant (maladaptive) coping.

Studies utilizing structural equation modeling (SEM) have demonstrated that MCOP either fully or partially mediate the relationship between perfectionism and depression in adults [e.g., Ref. (29–31)]. However, no known published research has examined the mediating role of coping on the depression–perfectionism relationships in children.

Exposing the interactions of MCOP, perfectionism, and depression may have implications for the development of effective treatment and prevention of depressive disorders, particularly given Blatt and colleagues’ findings in a landmark study [see Ref. (32)] that perfectionism can limit the effectiveness of otherwise efficacious treatment for depression in adults. Interventions addressing depressive symptoms in adults with high levels of perfectionism have only been efficacious when directly targeting perfectionistic behaviors and cognitions [e.g., Ref. (33–35)].

Although there are no known evidenced-based treatments for children that specifically target perfectionism, preliminary research indicates that CBT-based interventions aimed at preventing and reducing depression symptoms may also influence perfectionism. Essau et al. (36) reported significant improvements in both SOP and SPP as well as coping and depression symptoms as a result of a randomized controlled trial of the manualized FRIENDS program administered to a large community sample of 9- to 12-year-old children. A randomized controlled study by Nobel et al. (18) in children with subclinical levels of anxiety and depression also observed reductions in depression and SOP. As there were no significant differences between treatment and control groups at post-test, conclusions regarding treatment efficacy were limited. A study by Dry (37) found positive changes over time in depression levels of 8- to 11-year-old children with high levels of perfectionism in an open trial of the CBT-based Aussie Optimism Positive Thinking Skills program. However, the absence of a control group limited inferences that could be made about treatment efficacy.

The purpose of this study was to address specific limitations of the aforementioned research and examine the interrelationships between coping strategies, perfectionism, and prodromal depression symptoms in the context of a preventative treatment for pre adolescents. It was specifically hypothesized that:

H1: Pre-test perfectionism (SOP and SPP) would moderate the intervention effect on depression scores, such that children with high-perfectionism scores would benefit less from the intervention;
H2: At pre-test, MCOP would partially mediate the relationship between perfectionism (SOP and SPP) and depression symptoms, such that individuals with high levels of perfectionism would score more highly on MCOP and depression symptoms. It was expected that this mediation relationship would be stronger for SPP. As researchers reported gender differences in rates of SOP and SPP (28), in coping strategies [e.g., Ref. (38)] and rates of depression symptoms (11), it was expected that gender would be a covariate; and
H3: Adaptive coping would partially mediate the therapeutic effect of the intervention on depressive symptoms.

## Materials and Methods

### Participant characteristics

The sample comprised 541 children (49.5% males, 50.5% females) aged 8–12 years (*M* = 9.72, *SD* = 1.06) from a predominantly Australian background (87.7%). Children attended years 4 (56%) and 5 (44%) at 10 Catholic primary schools from lower to middle socioeconomic ranked suburbs within the Perth Metropolitan region, WA, Australia. Demographic information was derived from questionnaires completed by 77% of parents.

### Sampling procedures

This study’s sample was drawn from a larger longitudinal study, the 2012 Aussie Optimism Positive Thinking Project (AO-PTS). AO-PTS researchers randomly selected schools from a pool of 30 co-educational independent schools that satisfied a double stream per year minimum class size requirement and an absence of prior treatment programs. Schools were paired according to the socioeconomic status measure of the school’s postcode and class size and then randomly assigned to either the intervention or control conditions. The study sample comprised the first five intervention and control groups for which pre- and post-test data collection had been completed at the time of analysis.

### Research design

A cluster randomized controlled trial was undertaken to investigate the relationships between a universal intervention program and pre and post measures of perfectionism, coping, and depression. The endogenous variables were children’s depression symptoms, maladaptive, and adaptive coping strategies; the exogenous variable was children’s pre-test perfectionism.

### Sample size

*A priori* power analysis using Kline (39) recommendations of a 20:1 sample size-to-parameter ratio was used for estimating the sample size for the Structural Equation models. Based on a maximum number of 13 parameters, the obtained sample size of 541 well exceeded the *a priori* determined sample size of 260.

### Instruments

#### Intervention

*The Aussie Optimism: Positive Thinking Skills Program* (40) is a CBT-based universal intervention aimed at preventing internalizing disorders in preadolescents. The manualized program was administered weekly to students over 1 h session for a period of 10 weeks by teachers specifically trained in the intervention. The 10 treatment modules included the following age appropriate content: positive activity scheduling; identifying feelings in self and others; situational impact on feelings; overcoming fear; becoming aware of thoughts and their connection to feelings; awareness of helpful and unhelpful thinking; challenging unhelpful thinking and adopting more positive thoughts, and consolidation of principles learned. A previous trial of the universally administered classroom-based program demonstrated reductions in depressive symptoms (41).

#### Measures

The following lists the self-rated measures relevant to this project administered as part of the larger AO-PTS assessment battery.

*The Child and Adolescent Perfectionism Scale* (Flett et al., Unpublished)^1^ is a widely used 22-item measure to assess children’s self-oriented and SPP tendencies. The items have been worded to be suitable for children of a 3-year level reading age and are self-rated on a 5-point scale (Flett et al., Unpublished)^1^. Flett et al. (Unpublished)^1^ demonstrated sound reliability and test–retest reliabilities for the full scale rates and both subscales, and discriminant and concurrent validity. Cronbach’s alpha rates in this sample were α = 0.84 for SPP and α = 0.73 for SOP, the latter being somewhat marginal for the purposes of analysis and considerably lower than that reported by Flett et al. (α = 0.85).

*The Children’s Depression Inventory* [*CDI;* (42)] is a 27-item scale frequently used in research and clinical applications. Children are required to select one of three statements that most closely reflect their dominant emotional state in the previous 2 weeks. Statements indicate absent, mild, or strong depression symptoms. Item No. 19 (evaluating suicide ideation) was removed from the questionnaire due to concerns raised by school principals (43). The abbreviated scale in this sample yielded a Cronbach’s alpha reliability rate of α = 0.89. Kovacs (42) demonstrated discriminant and concurrent validity for the scale and reported sound test–retest reliability rates, ranging from *r* = 0.66–0.83 at 2–4 weeks.

*The Coping Scale for Children and Youth* [*CSCY;* (44)] is a 29-item 4-point scale in which children rate how frequently they use a particular coping behavior in response to a problem. The scale differentiates between adaptive coping styles of assistance seeking and cognitive behavioral problem solving, and MCOP styles of cognitive avoidance and behavioral avoidance. Essau et al. (36) reported an alpha reliability of α = 0.84. The current sample yielded Cronbach’s alpha of 0.82 and 0.85 for the adaptive and maladaptive subscales, respectively. This instrument was reported to have sound test–retest reliability ranging from *r* = 0.73 to 0.81 (44).

### Data collection procedure

Ethics approval was obtained from both the Curtin University Human Ethics Committee and the Catholic Education Commission. Once school principals had agreed to participate, written informed consent was sought from parents and children. Trained experimenters, who were blind to the treatment mode, administered the battery of measures in accordance with the AO-PTS protocol manual to participating children in classroom groups during class time, reading aloud all questions to minimize differences in reading ability. Where possible a teacher was present at testing to ensure duty of care was maintained. Children were rewarded with a sticker and pencil or eraser for participating. After data entry, the AO-PTS research team identified children who scored highly on the CDI [above 17, Ref. (42)]. Parents were notified accordingly offered referrals for appropriate psychological support for their children.

Testing was conducted before and after the 10-week intervention, or in the case of the control group after an equivalent lapse of time. Data collection occurred between 17 September 2010 and 14 December 2010 for the intervention schools and between 3 August 2011 and 6 December 2012 for the control schools. The difference in timing was due to difficulties in recruiting control schools.

Records of attendance rates and treatment compliance were maintained by teachers but remained incomplete to date. Although data were analyzed on intent-to-treat basis, interpretation of treatment effects should be made with caution.

### Data analysis

Hypotheses were tested by means of SEM using LISREL v. 8.80 software (45). An advantage of SEM is the ability to input alpha reliabilities to reflect measurement error of measures used (46). Assumption and prerequisite testing for the SEM were undertaken using Statistical Package for the Social Sciences (SPSS) version 19.0 software.

## Results

### Participant flow

Parental and student consent for participation was obtained for 650 (50.15%) of 1,296 eligible students. Twenty-two students (11 in each condition) were absent at pre-test, while 43 students (16 in the intervention group, 27 in the control group) were absent at post-test. Consent was withdrawn for 21 students. Cases with more than 15% of missing values over a single variable amounted to 14 at pre-test and an additional 15 cases at post-test (1 of whom withdrew consent). After deleting cases of consent withdrawal, partial wave non-response and cases with excessive missingness, 541 cases were available for analysis.

### Screening and missing values

Missing data extended to 6.4% of responses, with 101 out of 164 variables recording more than 5% missing values (maximum 9.7%). Little’s MCAR test was significant with χ^2^(34,696, *N* = 650) = 36,460.88, *p* < 0.001, indicating that data were not missing completely at random. Prior to pairwise deletion, the 88 cases of partial or complete wave non-response were screened for elevations on the CDI (scores above 17). Fourteen children (six in the intervention and three in the control group) were identified at being at-risk for depression, thus deletion of these cases may have biased results.

However, cases of partial or full wave non-response needed to be deleted pairwise as the SEM is not robust to missingness (39). For the remaining 541 cases, where missingness was <15% per measure, missing values were replaced using a maximum likelihood estimation, expectation maximization (EM), in SPSS (Version 19). While values were not missing at random, the true cause of missingness is usually only moderately correlated with outcomes and therefore replacing missing values with EM would be of minimal consequence to estimates (47, 48).

### Descriptive statistics

Table 1 shows the mean of total scores and SDs in addition to the range of scores for all outcome variables for both waves of testing according to test condition. The mean scores for depression symptoms in both groups, while higher in the control group, were well below the clinically significant cut off levels (>17), which would be consistent with a healthy community sample. Despite low means scores, 54 children were identified as being at-risk for depression (24 in the intervention group and 30 in the control group) at pre-test, while 50 children were identified at-risk for depression (27 in the intervention group and 23 in the control group) at post-test. Mean scores for SPP and SOP were comparable to those reported by Huggins et al. (19), Flett et al. (Unpublished)^1^, and Flett et al. (14).

**Table 1:** **Scores on outcome measures for control and intervention groups at both waves of testing**.

Measure	Mean (SD)	Range	95% CI	Mean (SD)	Range	95% CI

	Pre-test			Post-test		
**INTERVENTION**						
CDI	7.06 (6.40)	0–33	[6.35, 7.77]	6.18(6.92)	0–40	[5.41, 6.95]
CSCY-A	2.65 (0.54)	1.25–3.92	[2.58, 2.71]	2.62 (0.53)	1.08–3.92	[2.56, 2.68]
CSCY-M	2.31 (0.53)	1.06–4.00	[2.25, 2.36]	2.30 (0.57)	1.00–4.00	[2.24, 2.37]
SPP	27.24 (7.68)	10–50	[26.39, 28.10]	26.11 (8.09)	10–50	[25.21, 27.01]
SOP	37.94 (6.60)	12–60	[37.21, 38.67]	36.81(7.18)	12–60	[36.01, 37.61]
**CONTROL**						
CDI	8.93 (8.09)	0–40	[7.86, 10.00]	7.56 (7.39)	0–46	[6.59, 8.53]
CSCY-A	2.62 (0.56)	1.04–3.93	[2.55, 2.70]	2.55 (0.61)	1.00–4.00	[2.47, 2.63]
CSCY-M	2.32 (0.50)	1.06–3.9	[2.25, 2.35]	2.20 (0.51)	1.00–3.76	[2.13, 2.27]
SPP	27.06 (7.98)	10–48	[26.01, 28.10]	25.47 (8.63)	9–50	[24.34, 26.60]
SOP	38.38 (6.67)	21–58	[37.50, 39.25]	37.10 (7.56)	12–60	[36.11, 38.09]

*Intervention group A = 314, control group N = 227*.

*CI, confidence interval; CDI, child depression inventory; CSCY, coping scale for children and youth; A, adaptive coping; M, maladaptive coping; SPP, socially prescribed perfectionism; SOP, self-oriented perfectionism*.

*Missing values were deleted list wise where missingness was >15% per variable per case, other missing values were EM replaced*.

### Assumption testing

The data were screened for the assumptions of univariate and multivariate normality, linearity, absence of multicollinearity and singularity of covariance, and symmetrical distribution of residuals (46). Violations to univariate normality were observed on CDI scores, which were positively skewed. A test of multivariate normality for continuous variables was significant with χ^2^ = 280.06, *p* < 0.001. A violation to normality necessitated the computation of a Spearman correlation matrix for input into the structural equation model (49). Although multivariate outliers were detected for 12 cases, exceeding the critical Mahalanobis chi-square values [χ^2^(16) = 36.12, *p* < 0.001], these were retained as correlation matrices were unaffected.

### Prerequisite analyses

As hypotheses H1 and H3 were examining relationships between variables in the context of an intervention effect, the presence of an intervention effect on outcome variables needed to be established. This was achieved with the use of the Generalized Linear Mixed Models (GLMM) procedure, which produces a multi-level mixed effects linear regression. This is the statistical method of choice for examining intervention effects in data that are multi-level in nature, has unequal group sizes (intervention and control), and in which outcome variables correlate with each other between tests (50). It also makes adjustments to address violations to normality. To take into account the nested nature of the data, participants, year, and schools were controlled for as random factors in the model. Fixed factors included one categorical fixed effect (group: intervention, control), and one ordinal fixed effect (time: pre, post).

Results of the GLMM, summarized in Table 2, demonstrate that there was a significant, albeit small, interaction with mean scores for all measures over time. While there was a significant interaction of treatment mode and reductions on depression and MCOP measures, these were attributed to small but significant reductions in the control group [CDI: *F*(1,1078) = 8.13, *p* < 0.01, η^2^ = 0.01; and MCOP *F*(1,1078) = 10.84, *p* < 0.01, η^2^ = 0.01], indicating changes in observed mean scores were not due to the intervention. The absence of any observed intervention effects meant that the prerequisite for testing hypotheses 1 and 3 was not met.

**Table 2:** **Fixed effects estimates for interactions with time and intervention mode**.

Source	*F*	Significance	Effect size ***η***^2^
**CDI**			
Group	4.90	0.01*	<0.00
Time	8.5	0.03*	0.01
Group × time	0.42	0.00**	<0.00
**SPP**			
Group	0.23	0.64	
Time	29.41	0.00***	0.03
Group × time	0.81	0.37	
**SOP**			
Group	0.41	0.52	
Time	16.16	0.00***	0.01
Group × time	0.08	0.78	
**ADAPTIVE COPING**			
Group	0.09	0.77	
Time	4.96	0.03*	<0.00
Group × time	1.11	0.29	
**MALADAPTIVE COPING**			
Group	0.38	0.54	
Time	5.54	0.02*	<0.00
Group × time	5.20	0.02*	<0.00

dfnumerator = 1, dfnominator = 1,078. *p < 0.05; **p < 0.01; ***p < 0.001.

For the remaining structural equation hypothesis, H2, a Spearman’s rank order correlation matrix was produced to confirm the presence of relationships between predictor and criterion variables, a prerequisite for testing mediation, and to examine presence of covariates (Table 3).

**Table 3:** **Spearman correlations of observed measures at pre-test**.

Variable	1	2	3	4	5	6	7	8	9
1. ICSEA	–
2. Gender	0.07	–							
3. Depression symptoms	−0.04	−0.17**	–						
4. Socially prescribed perfectionism	−0.14**	−0.14**	0.30**	–					
5. Self-oriented perfectionism	−0.10*	0.04	0.03	0.44**	–				
6. Adaptive coping-assistance seeking	0.03	0.21**	−0.12**	−0.16**	0.11*	–			
7. Adaptive coping-cognitive behavioral problem solving	0.03	0.13**	−0.10*	0.05	0.15**	0.45**	–		
8. Maladaptive coping-cognitive avoidance	−0.07	−0.08	0.13**	0.26**	0.14**	−0.02	0.34**	–	
9. Maladaptive coping-behavioral avoidance	−0.04	0.00	0.29**	0.34**	0.22**	0.13**	0.30**	0.56**	–

N = 541. ICSEA, index of community socio-educational advantage (54). *p < 0.05, two-tailed; **p < 0.01, two-tailed.

Socially prescribed perfectionism scores correlated moderately (*d* = 0.63) and positively with depression symptoms and scores for MCOP strategies, while correlations with scores on assistance seeking were negative and weaker. SOP scores positively correlated with all of the coping scores however did not correlate with depression. Adaptive coping correlated weakly and negatively with depression symptoms, while MCOP correlated positively with symptoms of depression. The mediation criteria of correlations between depression, SPP, and coping were satisfied.

Gender was correlated with more than two of the observed variables, each of which measured at least two different latent variables, thereby indicating that it was likely to influence interactions among the latent variables in the pathway model and needing to be controlled for in the SEM. Subsequently, a partial correlation matrix controlling for gender was computed and entered into LISREL. Incidentally, there were no significant differences in correlations between the Spearman and gender controlled correlation matrices.

### Hypothesis testing

**Figure 1:**
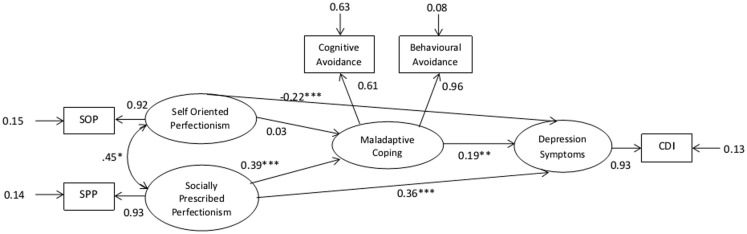
**Gender controlled saturated structural equation model at pre-test**. *N* = 541. *p < 0.05, two-tailed; **p < 0.01, two-tailed; ***p < 0.001.

**Figure 2:**
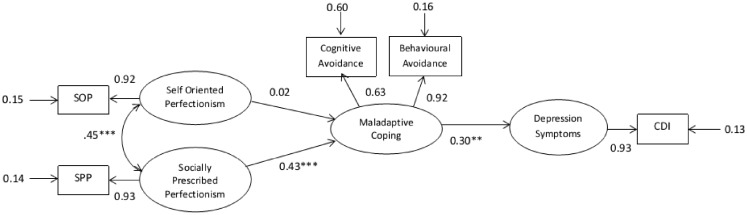
**Gender controlled mediator structural equation model at pre-test**. *N* = 541. *p < 0.05, two-tailed; **p < 0.01, two-tailed; ***p < 0.001.

The mediation hypothesis was tested by specifying pathways between latent variables (perfectionism, depression and coping) in LISREL to reflect the hypothesized relationships. In addition, measurement variables were specified for perfectionism (SOP and SPP), coping (maladaptive and adaptive), and depression (CDI). Measurement errors for subscales were derived from the Cronbach alpha rates from published studies and were entered into the SEM or computed by the model where no published data were available. Two competing structural models – a saturated model (Figure 1) with a maximum number of pathways and a nested, more parsimonious model testing mediation (Figure 2) – were then tested for fit and compared. Fit statistics for the two models are shown in Table 4.

**Table 4:** **Fit statistics for the measurement components of the saturated and mediator model**.

Model	***χ***^2^/df	Comparative fit index (CFI)	Non-normed fit index (NNFI)	Standardized root mean square residual (SRMR)	Root mean square error of approximation (RMSEA)
Saturated model	10.12/2 = 5.06	0.98	0.92	0.02	0.13 (90% CI: 0.04, 0.14)
Mediator model	41.95/4 = 10.49	0.93	0.81	0.05	0.13 (90% CI: 0.09, 0.16)

*χ^2^/df: Good Fit is indicated by a value <3 (39); CFI: Good Fit is indicated by a value ≤0.90; NNFI: Good Fit is indicated by a value ≥0.90; SRMR: Good Fit is indicated by a value ≤0.08 (55); RMSEA: Good Fit is indicated by a value ≤0.05, or a CI that encompasses this value (56)*.

A sandwich estimator was used to adjust the path coefficients’ SEs to control for the potentially inflationary effects of intra-school dependencies on the Type I error rate (51). Intra-school correlation coefficients were calculated by means of a 2 (year groups) × 6 (outcomes) ANOVA. Year groups accounted for 0.002–3.7% of variance on scores of output measures. *p*-Values for pathway coefficients were adjusted accordingly.

Path coefficients for predicted pathways were significant in both models with the exception of the SOP and MCOP path. Although the mediator model produced a reasonable fit, the statistics for the saturated model indicated a better fit. The fit statistics were significantly different with χ^2^diff = 31.83, df = 2, *p* < 0.001, confirming that the saturated model produced the superior fit and contributed to 16.5% of the variance in depression scores at pre-test.

The direct pathways from SOP and SPP to depression (DEP) on the better fitting model were both significant. The model was therefore further examined to determine whether conditions were met for mediation, which is whether MCOP partially mediated these relationships. Conditions required to meet mediation are described below.

*Condition 1*: DEP needs to be correlated with both SOP and SPP. The correlation matrix of latent variables presented below indicates that this condition is satisfied (Table 5).
*Condition 2*: The three component pathways that comprise the indirect effects (SOP → MCOP, SPP → MCOP, and MCOP → DEP) must all be significant. The *p*-values reported in Figure 2 indicate that this condition is satisfied for only two of the three component pathways (SPP → MCOP and MCOP → DEP).
*Condition 3*: The overall indirect effect from SPP to DEP via MCOP must be significant. The strength of the indirect effect is given by the product of its two component path coefficients; 0.39 multiplied by 0.19 equals −0.074, which is significantly >0 (*z* = 3.10, *p* = 0.002). The indirect effect from SPP to DEP via MCOP is therefore significant, which satisfies Condition 3.
*Condition 4*: The strength of the direct pathway from SPP to DEP (0.36) must be significantly less than the strength of this pathway after removing MCOP from the model (0.43). A *z* value of 1.96 or greater is required for significance at the 0.05 significance level. However, the computed *z* value was 0.84 and therefore suggests that there is no significant difference between the two path coefficients. Unfortunately, the fourth condition for partial mediation is *not* satisfied.

**Table 5:** **Correlation matrix of latent variables**.

	SOP	SPP	MCOP	DEP
SOP	1			
SPP	0.525, *p* < 0.001	1		
MCOP	0.236, *p* < 0.001	0.408, *p* < 0.001	1	
DEP	0.008, *p* = 0.871	0.317, *p* < 0.001	0.282, *p* < 0.001	1

To conclude, SPP had both a direct and indirect effect (via mcop) on depression. The indirect effect, however, does not reflect partial mediation.

## Discussion

The research aims of this study were to examine whether perfectionism limited the effectiveness of an intervention aimed at preventing depression in preadolescents, whether coping mediated the relationship between perfectionism and depression at pre-test, and whether increases in coping facilitated the effectiveness of the intervention.

The absence of an observed intervention effect rendered hypothesis 1 and 3 untestable. Considering that a previous trial of the AO-PTS intervention found significant effects in a larger sample of children where mean CDI scores were approximately 1 SD higher (*M* = 12.21, *N* = 467) than those of the current sample, a key factor limiting the observation of an intervention effect in this sample was an apparent floor effect of scores for depression symptoms, potentially exacerbated by wave attrition of a disproportionate number of children with elevated scores for depression in the control group. Additionally, as children with elevated scores for depression were offered referrals for psychological assistance, any external support received may have contributed to the observed reduction in depression scores. It is possible that this contributed to the observed pre–post-test reductions in the control group, which comprised a higher number of at-risk children who may have received psychological help, thereby mitigating any treatment effect. Additional factors limiting observation of an intervention effect included potential temporal effects due to differences in timing of testing for control and intervention groups and potential inconsistencies in treatment compliance by teachers (this could not be determined due to incomplete records of treatment fidelity and child attendance rates for some schools).

It was predicted that at pre-test, MCOP would partially mediate the relationship between perfectionism and depression symptoms and that this mediation relationship would be stronger between depression and SPP. Results confirmed that gender was a covariate, influencing several of the outcome measures. The SEM found that SPP had a direct and indirect association with depression but that coping was not a mediator. This finding suggests that while children with high levels of SPP tend to employ more dysfunctional coping strategies to manage stress, thereby contributing to depression symptoms, other perfectionistic tendencies, such as self-criticism and rumination, over failure to meet others’ perceived expectations have an independent and stronger relationship with depression. These findings are contrary to SEM research in adults, which demonstrated more clearly that avoidant coping mediated the relationship between perfectionism and depression [e.g., Ref. (30, 52)]. Wei et al.’s (30) more sophisticated longitudinal design demonstrated that maladaptive perfectionism and coping not only contributed to depression but also influenced each other at different points in time. Dunkley and Blankstein’s (52) mediation model was demonstrated in the context of daily hassles and distress. It is feasible that the more complex models of coping and perfectionism tapped into the latent variables more effectively. Zhang and Cai (31) claimed partial mediation in a simpler cross-sectional model; however, the patterns of association observed in their sample of young adults were similar to those observed in this sample with direct pathways relatively stronger compared to indirect pathways, casting doubt over their claim of partial mediation.

The SEM demonstrated a small but significant negative association between depression and SOP, indicating that there may be protective factors associated with this dimension of perfectionism. SOP also correlated positively with cognitive behavioral problem solving, indicating that children who are more self-motivated to achieve may be less likely to suffer from depression. However, this conclusion would be inconsistent with findings in adult and child literature by Nobel et al. who found a positive association between SOP and depression. This contradiction may be due to differences in levels of distress in the sample, with Nobel et al.’s sample showing higher levels of depression symptoms. Hewitt et al. (16) and O’Connor et al. (17) observed that stress moderated the association between perfectionism (including SOP) and depression in adolescent youth. This would suggest that maladaptive cognitions associated with SOP, such as rumination over mistakes, procrastination, and self-criticism, may only be triggered in the presence of acute stress. Lewis and Frydenberg (25) observed that adolescents who implemented adaptive coping strategies also used MCOP strategies, concluding that adolescents may adopt MCOP strategies when other strategies have failed. Others have suggested that avoidance strategies may be the default choice in times of distress (25, 44).

Consistent with research by Flett et al. (28), SPP scores showed a moderate and positive association with MCOP, while SPP was negatively associated with adaptive coping, suggesting that children of this younger age group with tendencies to critically evaluate themselves against perceived high expectations tend to use more behavioral and cognitive avoidance strategies to deal with stressors.

### Strengths and limitations

The strengths of this study included a sound methodological approach comprising a randomized control design, standardized testing procedures, a large sample size ensuring a sufficiently powered statistical analysis, and sophisticated statistical methods to assess the interaction of outcome variables, which were able to account for measurement error, group inequivalence, and intra-class dependence.

Because the mediation hypothesis was tested cross-sectionally, inferences regarding causal relationships implied in the model are limited as the temporal sequencing of perfectionism, coping, and depression was not demonstrated (53). Ideally, mediation is tested longitudinally over three points in time without potential interference of an intervention. As data were unavailable for three separate time points, it was decided to examine meditational interactions concurrently at pre-test, combining intervention and control subjects to maximize sample size and thereby increasing statistical power. Other limitations included reliance on self-reports of children and poor reliability rates on two of the subscales of the CSCY, which may have limited observed relationships (although error rates were accounted for in the SEM). Finally, the generalizability of results is limited as participating children were from predominantly Catholic middle income families.

### Practical and theoretical implications

This study extended on a very limited body of research demonstrating links between MCOP and perfectionism and presenting novel research results of SEM of the interrelationships between socially prescribed and SOP, coping, and depression. The pathway model identified provides an insight into the interactions of these variables in asymptomatic children, and also provides a more accurate analysis (by accounting for measurement error) of relationships between perfectionism and depression, which has previously been examined primarily through means of correlational studies.

It is recommended that future research examine these interrelationships in a longitudinal study to demonstrate temporal sequence in a causal model. It is also advised that a coping measure is used, which has demonstrated strong internal consistency across all subscales to improve construct validity. Further research validating the CSYC for use in a younger sample should be considered. Future examination of the relationship between SOP coping and depression in a diathesis-stress model may highlight maladaptive traits of SOP in healthy samples, which could further inform the formulation of preventive strategies. As suggested by Pincus and Friedman (26), such preventative interventions may need to include not only an increase in adaptive coping skills but also an increased awareness of the effects of using less productive coping strategies in times of stress. Future research testing for moderation of perfectionism on efficacious treatments in children is warranted as is an investigation into whether improving coping skills would buffer against the effects of perfectionism on depression.

## Conclusion

The study through sophisticated SEM supported that children with a tendency to evaluate themselves critically against the perceived unrealistically high expectations of others tend to report higher levels of depression. This influence is predominantly due to the maladaptive cognitions associated with perfectionism, and to a lesser extent, as a result of an increased tendency of these children toward to use of ineffective behavioral and cognitive avoidance strategies to cope with stressors.

Furthermore, the findings that associations between perfectionism, MCOP, and depression are present even in children who do not necessarily present clinical levels of depression symptoms could be useful in informing universal programs to prevent adverse mental health outcomes in children.

## Conflict of Interest Statement

The authors declare that the research was conducted in the absence of any commercial or financial relationships that could be construed as a potential conflict of interest.

## Footnotes

^1^Flett GL, Hewitt PL, Boucher DJ, Davidson LA, Munro Y. The Child-Adolescent Perfectionism Scale: Development, Validation, and Association with Adjustment (2000), (Unpublished).
